# Ovarian Hemangioma With Stromal Luteinization

**DOI:** 10.7759/cureus.29438

**Published:** 2022-09-22

**Authors:** Sara Correia, Maria J Oliveira, Xiaogang Wen

**Affiliations:** 1 Endocrinology, Centro Hospitalar de Vila Nova de Gaia/Espinho, Vila Nova de Gaia, PRT; 2 Anatomical Pathology, Centro Hospitalar de Vila Nova de Gaia/Espinho, Vila Nova de Gaia, PRT

**Keywords:** luteinization, menopause, hyperandrogenism, hirsutism, ovarian hemangioma

## Abstract

Ovarian hemangiomas are generally asymptomatic. However, associated stromal luteinization, reported in some cases, may lead to the development of hyperandrogenic syndrome. We report the case of a 61-year-old female referred to the endocrinology outpatient clinic for hirsutism, hair loss, and deepening of the voice tone. The investigation showed high serum testosterone and normal dehydroepiandrosterone sulfate (DHEAS). Normal ovaries were observed in the initial transvaginal ultrasound, but an abdominal-pelvic nuclear magnetic resonance imaging (MRI) identified a solid mass in the right ovary. The patient underwent surgery, and pathological examination revealed a capillary-type ovarian hemangioma with stromal luteinization. After the surgery, clinical and analytical response was very favorable.

## Introduction

During menopause, ovaries maintain some residual hormonal activity. There is an imbalance between estrogens and circulating androgens, in favor of the latter, amplified by the decrease in the concentration of sex hormone-binding globulin (SHBG), which can lead to the appearance of clinical signs of hyperandrogenism [1,2]. However, when hirsutism has a sudden onset and rapid progression and is accompanied by other signs of virilization, such as alopecia, masculinization of the voice, and clitoromegaly, the suspicion of a virilizing ovarian tumor or androgen-producing adrenal tumor increases [1,2]. The phenotype alone does not make it possible to discriminate between a tumoral and non-tumoral hyperandrogenic cause.

Ovarian hemangiomas are benign, rare, and usually small-sized tumors, so their detection can be a challenge. Stromal luteinization, reported in some cases in the literature, is an uncommon process and is responsible for the secretion of androgens, which can lead to hyperandrogenic syndrome. Its pathogenesis is unknown and controversial [3,4].

## Case presentation

A 61-year-old Caucasian female with a history of gastritis and colic diverticulosis was referred to the outpatient endocrinology clinic because of hirsutism and high levels of testosterone. She complained of progressive hirsutism, hair loss, masculinization of the voice, and an increased weight of 3 kg in the previous three months. She denied vaginal blood loss. She was medicated on lansoprazole 30 mg daily, cholecalciferol 25,000 IU once a month, and estriol 1 mg/g vaginal cream applied daily since five years ago. Her gynecological and obstetric histories are as follows: menarche at 14 with regular cycles, two pregnancies and two eutocic births, oral contraceptives from 35 to 54 years old, and menopause at 55 years old. Her family history was irrelevant. Her physical examination results are as follows: body mass index of 28 kg/m^2^; a low tone of voice; hirsutism in the face, chest, and abdominal region (11/36 points on the Ferriman-Gallwey score); enhanced muscle development at the scapular level and deltoid region; and rarefaction of the scalp in the frontoparietal region. There was no evidence of acanthosis nigricans or cushingoid phenotype. Her blood pressure was 126/72 mmHg. Her abdominal examination did not reveal masses or organomegaly. Gynecological inspection showed clitoromegaly. Blood count, fasting blood glucose, ionogram, and renal and liver function tests were normal. Her lipid profile was deranged. The hormonal study revealed very high total and free serum testosterone, favoring an androgen-secreting tumor (Table 1).

**Table 1:** Analytical study on admission SHBG: sex hormone-binding globulin; DHEAS: dehydroepiandrosterone sulfate; FSH: follicle-stimulating hormone; LH: luteinizing hormone; ACTH: adrenocorticotropic hormone; TSH: thyroid-stimulating hormone; LDL: low-density lipoprotein; HDL: high-density lipoprotein; CA 125: cancer antigen 125; CEA: carcinoembryonic antigen

Parameters	Results	Reference values
Total testosterone	4.20 ng/mL	0.06-0.82 ng/mL
Free testosterone	8.40 pg/mL	0.1-1.80 pg/mL
SHBG	35.6 mmol/L	20-130 mmol/L
DHEAS	47.4 µg/L	18.9-205 µg/L
Delta 4-androstenedione	5.3 ng/mL	0.5-4.7 ng/mL
17-Hydroxyprogesterone	13.3 ng/mL	0.95-5 ng/mL
FSH	10.38 mUI/mL	25.8-134.8 mUI/mL
LH	7.80 mUI/mL	7.7-58.5 mUI/mL
Estradiol	72.8 ng/L	5-7.97 ng/L
Serum cortisol (eight hours)	23.9 µg/dL	6.2-19.4 µg/dL
ACTH (eight hours)	11.4 pg/mL	0-46 pg/mL
Urinary cortisol (24 hours)	37.3 µg/24 hours	20-90 µg/24 hours
TSH	1.85 uUI/mL	0.27-4.2 uUI/mL
Prolactin	11.8 ng/mL	4.71-23.3 ng/mL
Total cholesterol	225 mg/dL	120-200 mg/dL
LDL cholesterol	148 mg/dL	3-100 mg/dL
HDL cholesterol	56 mg/dL	55-120 mg/dL
Triglycerides	106 mg/dL	<200 mg/dL
CA 125	7.7 U/mL	0-35 U/mL
CEA	1.6 ng/mL	0-4.3 ng/mL
Alpha-fetoprotein	3.84 UI/mL	0.5-5.8 UI/mL

**Figure 1:**
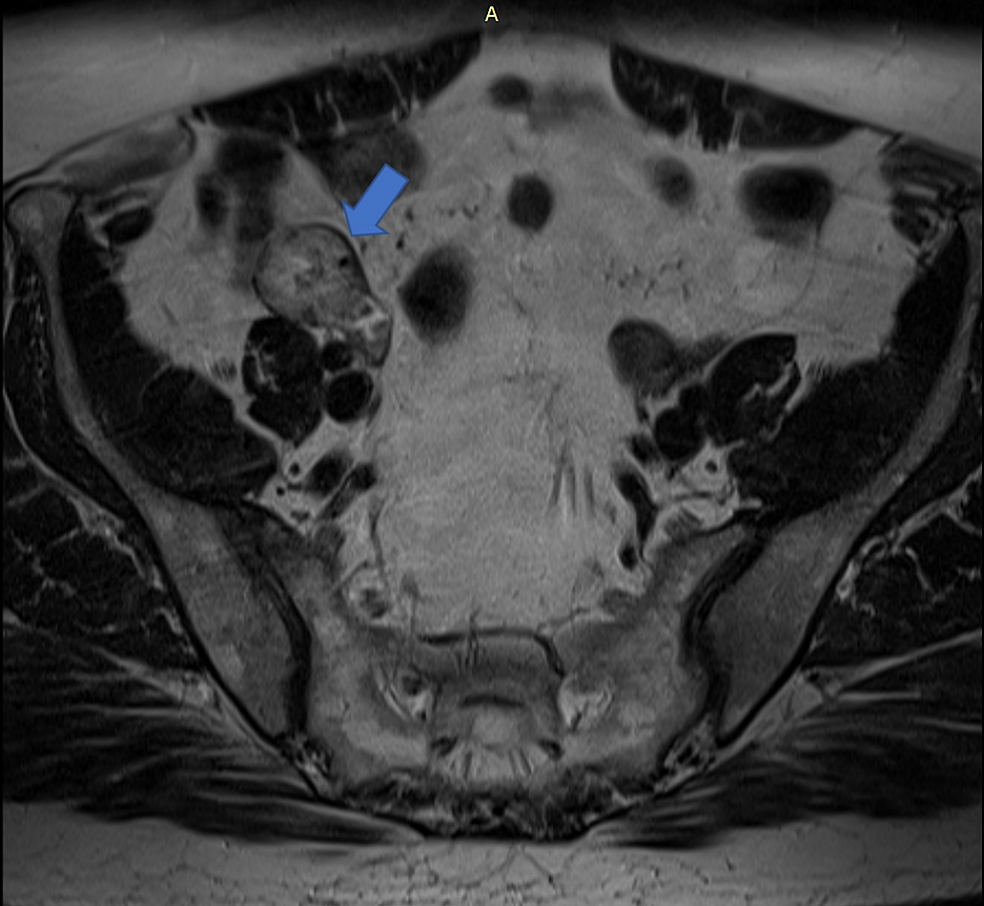
Pelvic magnetic resonance imaging A solid mass measuring 37 × 2 mm was relatively well defined in the right ovary (T2) (blue arrow).

The first two ultrasound scans, the endovaginal route and the other through the abdominal route, were normal. Nevertheless, given the strong suspicion of ovarian origin, an abdominal-pelvic nuclear magnetic resonance imaging (MRI) was performed, which identified a solid mass measuring 37 × 20 mm, relatively well defined in the right ovary (Figure 1), and 13 mm adenomas in both adrenals. Lymphadenomegalies or changes in other organs were not detected.

The ultrasound was repeated, but it was not possible to visualize the ovaries endovaginally. However, the abdominal approach allowed the identification of a solid mass in the right ovary. The left ovary was apparently normal. There was no evidence of ascites. Given the high value of 17-alpha-OH-progesterone (17-OHP), a genetic study was carried out that showed a variant in heterozygosity in the CYP21A2 gene, variant c.844G> T (p.Val282Leu), exon 7, which excluded the diagnosis of congenital adrenal hyperplasia. Given the adrenal incidentalomas, she underwent screening for pheochromocytoma and Cushing’s syndrome: urinary metanephrines were normal, and 1 mg dexamethasone suppression test = 2, pointing to a possible autonomous secretion of cortisol. Spironolactone was started at 100 mg, with a progressive dose increase up to 200 mg/day, and finasteride 5 mg, without signs of clinical improvement. Considering the suspicion of virilizing ovarian tumor, the patient underwent a total hysterectomy and laparoscopy bilateral annexectomy, which revealed isolated ovarian involvement. Peritoneal lavage was negative for neoplastic cells. The procedure was uneventful, and the patient was discharged on the fourth day after surgery. Five months after the surgery, the androgens and lipids returned to normal (Table 2), and the virilization signs regressed within five months.

**Table 2:** Analytical study five months after surgery SHBG: sex hormone-binding globulin; FSH: follicle-stimulating hormone; LH: luteinizing hormone; LDL: low-density lipoprotein; HDL: high-density lipoprotein

Parameters	Results (after the surgery)	Reference values
Total testosterone	0.09 ng/mL	0.06-0.82 ng/mL
Free testosterone	0.70 pg/mL	0.1-1.80 pg/mL
SHBG	102 mmol/L	20-130 mmol/L
17-Hydroxyprogesterone	2.4 ng/mL	0.95-5 ng/mL
FSH	47.79 mUI/mL	25.8-134.8 mUI/mL
LH	33.9 mUI/mL	7.7-58.5 mUI/mL
Estradiol	<5 ng/L	5-7.97 ng/L
Total cholesterol	144 mg/dL	120-200 mg/dL
LDL cholesterol	69 mg/dL	3-100 mg/dL
HDL cholesterol	65 mg/dL	>45 mg/dL
Triglycerides	51 mg/dL	<200 mg/dL

**Figure 2:**
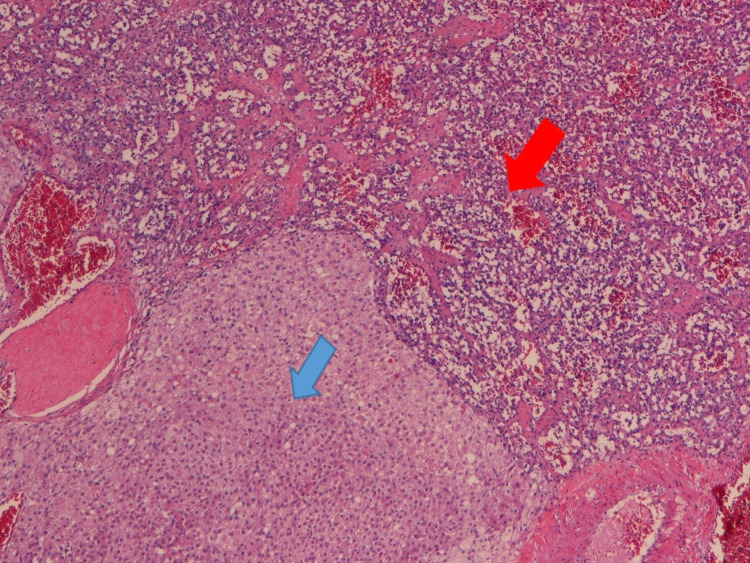
Histological image Anastomosing hemangioma of the ovary (red arrow) with stromal luteinization (blue arrow).

Vasomotor symptoms appeared again after cirurgy, with “hot flashes” and sweating. On histological examination, a vascular neoplasm was observed in the right ovary, with a vaguely lobular arrangement, consisting predominantly of capillary vessels of variable caliber and, in multiple areas with an anastomosing pattern, large vessels and a thickened wall without cytological atypia. A ring was identified around the vascular lesion, formed by the proliferation of stromal cells of the ovary (inhibin A +, PLAP -), with Leydig cell morphology and others with luteinized aspect, without cytological atypia. CA 125 was negative. The final histopathological diagnosis was anastomosing ovarian hemangioma with stromal luteinization (stromal cell hyperplasia) (Figure 2).

No pathological changes were found in the left ovary, fallopian tubes, and myometrium. Currently, the patient is asymptomatic, and no additional treatments were necessary.

## Discussion

The etiological diagnosis of hyperandrogenism can be a real challenge. During menopause, in the presence of virilization signs, the hypothesis of an androgen-secreting tumor should be considered [5,6]. The investigation should reconcile the clinical history, analytical study, and imaging examinations [1,2,6]. Hyperandrogenism is associated with an increased incidence of dyslipidemia, insulin resistance, arterial hypertension, and cardiovascular disease, with significant implications for the psychological well-being of the patient [1,5,7]. However, there is not always a correlation between the levels of androgens and the intensity of clinical manifestations [7]. In the present case, hormonal analyses were favorable to an ovarian cause. Usually, endovaginal ultrasound is the most used method for the identification of ovarian changes; however, it may not be diagnostic [6]. The reduced dimensions of some tumors, the experience of the operator, and the low acuity of the ultrasound may be limiting factors for its detection [6]. Given the high degree of suspicion, the performance of an abdominopelvic MRI allowed the identification of a mass in the right ovary.

Ovarian hemangiomas associated with stromal luteinization, reported in some cases, may give rise to the development of hyperandrogenic syndrome [8,9]. In this regard, two hypotheses have been suggested: luteinization either resulting from a reactive process or is a triggering factor for the development of hemangioma [3,4]. Although the mechanisms that lead to stromal luteinization are not clear, it is known that luteinized stromal cells produce steroid hormones, especially androgens, and these can subsequently be converted into estrogens in the adipose tissue [10]. Hemangiomas can thus mimic stromal tumors of the sexual cord of the ovary, such as the Leydig cell tumor or an epithelial carcinoma of the ovary, when serum CA 125 is significantly elevated [11]. Surgical resection of the tumor is the preferred treatment [8], allowing the normalization of the hormonal profile and the resolution of symptoms. Elevated levels of 17-OHP can also be seen in cases of androgen-secreting ovarian tumors [12]. The normalization of 17-OHP after oophorectomy suggests a case of intratumoral 21-hydroxylase deficiency. A case of a virilizing tumor in a 64-year-old female with high levels of 17-OHP that normalized after tumor resection was previously reported [13]. Given the risk of concomitant gynecological neoplasia and hemangiomatosis, careful observation of the contralateral ovary and endometrium is essential [3].

The reappearance of vasomotor disorders after surgery seems to mimic what happens when menopause begins, which is explained by the resolution of hyperandrogenism and consequently the drop in estrogens by the aromatization process. The definitive diagnosis is given by histopathological evaluation and immunohistochemical analysis [7,8]. A correct diagnosis is essential to avoid radical surgical procedures and unnecessary adjuvant treatments [8].

## Conclusions

During menopause, the appearance of signs of virilization should prompt the investigation of virilizing ovarian tumors. Although unusual, ovarian hemangioma with stromal luteinization should be considered in the differential diagnosis of hyperandrogenism. A high index of suspicion is warranted because androgen-secreting ovarian tumors may be missed because of their small size.
